# Neuronal boost to evolutionary dynamics

**DOI:** 10.1098/rsfs.2015.0074

**Published:** 2015-12-06

**Authors:** Harold P. de Vladar, Eörs Szathmáry

**Affiliations:** 1 Center for the Conceptual Foundations of Science, Parmenides Foundation, Kirchplatz 1, Pullach 82049, Germany; 2 Institute of Biology, Eötvös University, Pázmány Péter sétány 1/C, Budapest 1117, Hungary; 3 TMTA-ELTE Theoretical Biology and Evolutionary Ecology Research Group, Pázmány Péter sétány 1/C, Budapest 1117, Hungary

**Keywords:** evolutionary dynamics, Hebbian learning, synaptic plasticity, Darwinian neurodynamics

## Abstract

Standard evolutionary dynamics is limited by the constraints of the genetic system. A central message of evolutionary neurodynamics is that evolutionary dynamics in the brain can happen in a neuronal niche in real time, despite the fact that neurons do not reproduce. We show that Hebbian learning and structural synaptic plasticity broaden the capacity for informational replication and guided variability provided a neuronally plausible mechanism of replication is in place. The synergy between learning and selection is more efficient than the equivalent search by mutation selection. We also consider asymmetric landscapes and show that the learning weights become correlated with the fitness gradient. That is, the neuronal complexes learn the local properties of the fitness landscape, resulting in the generation of variability directed towards the direction of fitness increase, as if mutations in a genetic pool were drawn such that they would increase reproductive success. Evolution might thus be more efficient within evolved brains than among organisms out in the wild.

## 1. Background

Many mechanisms of cognition, memory and other aspects of brain function remain unclear. It is acknowledged that associations build up by updating synapses between neurons that spike (nearly) synchronously to a given stimulus. In this way, some neuronal circuits can predispose or anticipate a response to similar stimuli by retrieving information stored in synaptic weights. These weights may in turn be systematically altered in a Hebbian fashion by successful anticipation or recognition activity. At the same time, given the multi-dimensional space of alternative neuronal circuits and spiking sequences, undirected random variation in circuitry and spiking are extremely unlikely to produce better solutions for each new problem.

The connectivity of the human brain is sparse where, roughly, 10^11^ neurons are estimated to connect through some 10^15^ synapses. Learning and cognition have been understood in terms of changes in associative weights on networks of fixed topology. However, the discovery that rewiring this network is not uncommon even in adult brains challenges the former views regarding the mechanisms of learning. This rewiring, known as structural synaptic plasticity (SSP), has been well documented experimentally [1,2]. However, neither the full consequences nor the central role of SSP have been fully clarified. Yet, it is not only reasonable but also supporting evidence exists that SSP can encode information [3]. Thus, associative weights and SSP are two mechanisms that have an effect on learning. These need not be mutually exclusive; rather, in this article we deal with the two types of plasticity: Hebbian synaptic plasticity (HP), resulting in differential association weights among neurons, and SSP, leading to different topologies of the neuronal networks. Both types of plasticity can act on any given circuit during learning.

Our knowledge about what determines the establishment of new synapses is still limited, especially regarding the sparseness and dimensions of the brain. Neither synaptic weights nor SSP explain on their own the causes of the existing circuitry variability that is associated with a particular stimulus. If trial solutions to a problem (such as learning or recognizing a pattern) rely on serial evaluations, SSP is a poor candidate mechanism, even for long-term learning. Under serial evaluations, the time for establishing new synapses would be prohibitively large to account for randomly testing connections among pairs of neurons.

Changeux [4], Changeux *et al*. [5] and Edelman [6] proposed a selectionist [7] framework for brain function. They noted that selection acts through preferentially reinforcing and stabilizing some synaptic patterns over others and through the elimination of dysfunctional neurons and neuronal connections. Although these ideas are correct, they are incomplete because they only consider the fate of initial topological variability in circuitry, thought to occur only during development. In their framework, selection acts on this standing variation, stabilizing functional circuits that remain unchanged throughout life, with later learning and problem solving resulting only from HP. In this sense, the role of selection is limited to establishing a functional neuronal network in the early stages. The ideas that we investigate in this article go beyond this view: we consider that selection of novel variation plays an active role in learning through life. In this paper, ‘learning’ refers to the storage of a desired or target pattern by creating associations among neurons in a circuit. We assume also that stored patterns can be retrieved.

Kilgard [8] proposed a verbal model that accounts for circuitry variation during learning periods. In his ‘expansion–renormalization model (ERM)’, he envisions that SSP accounts for such variation. The mechanism is as follows. When a cortical subnetwork is challenged by a novel task, new synapses are being generated in response, out of which only the functionally important ones are kept, while the obsolete ones are eliminated. This is like an iterated Changeux-type overproduction–selective stabilization mechanism and is being explicitly regarded as a Darwinian mechanism by the author. However, he fails to discuss particulars such as: what the true units of variation are and how this mechanism quantitatively acts. Precisely, these aspects are what complement selection in order to implement true evolutionary dynamics in the brain. In other words, our ideas are conceptually similar to Kilgard's, but we pin them down to specific ‘learning’ units and develop quantitative models to understand how this variability is generated and how it affects learning.

We note that there are at least two other sources of neuronal variability. On one hand, we have the variance in spiking patterns in any given neuronal network due only to the stochastic behaviour of neurons (cf. [9,10]). On the other hand, different neuronal circuits involved in a task may have different association weights and different activities due to HP. Selection is then able to act on the variation that is generated by the three mechanisms. We point out that the crucial one is SSP, but, as we will explain throughout this article, the three mechanisms play different roles in learning.

We assume that circuits that result in a suboptimal solution relative to the rest of the circuits not only receive less reward, but also are more likely to be ‘overwritten’ by transmitting the information in the form of synaptic weights and structure from other local complexes. During this transmission process, small variations are introduced to the new circuit through SSP. Iterating this mechanism results in the increase in the representation of the circuit that gives the best solution, gradually replacing other circuits until no better variants are further produced, and finally (and ideally) a solution is found.

**Figure 1.**
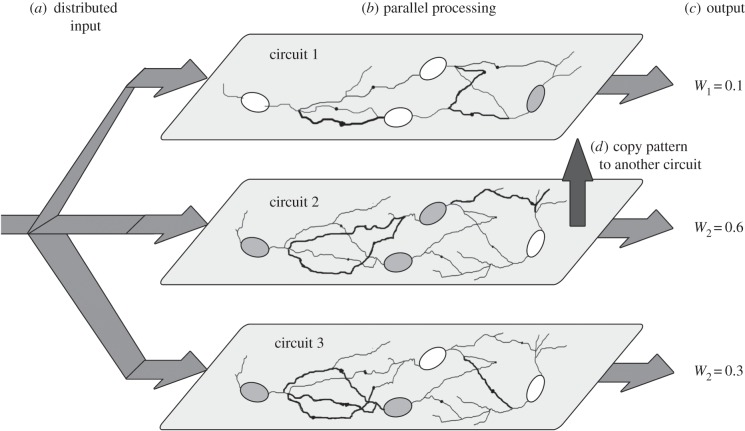
Replicative neurodynamics. (*a*) The input is fed into several local neuronal circuits. (*b*) Each of these circuits evaluates the input independently, thus trying in parallel distinct spiking patterns (represented by neurons in white and grey states), weights (line thickness) and topologies, and (*c*) producing distinct outputs with corresponding reward/fitness values *W*. (*d*) Circuits that result in higher fitness transmit their synaptic configurations to other circuits that performed poorly (connections among circuits are assumed to exist but are not displayed in the figure, and not explicitly modelled). This parallel evaluation is repeated until an optimal solution spreads across all circuits.

Our central aim is to understand how different neuronal complexes might evaluate possible solutions in parallel and thus compete to converge to an optimal result during learning (figure 1). For this, we put together all these verbal ideas into a quantitative framework.

We build up from local mechanisms of neural learning and set our problem at a time scale that allows us to follow whether neurons are found to be on or off. Each neuron is assumed to fire stochastically, but with a probability given by the input activity of other neurons in the complex. We will assume reinforcement learning, and, as in other works, employ simple measures such as distance between the output and the target. We emphasize that this is analogous to the gradient of a fitness landscape in evolution [11]. This analogy will allow us to tackle the problem with full force, partly by employing the models developed in evolutionary biology.

Despite the high level of abstraction of our approach, we acknowledge that an ultimate verification of our hypothesis needs to come from experimental neuroscience. However, at the moment, we intentionally avoid discussing molecular or physiological aspects, which, although essential to understand the problem experimentally, at this point would simply obscure understanding what we propose are the strategic means through which the brain works at the level we aim to describe.

### 1.1. Analogy with Darwinian evolution

As stated above, interpreting Neural Darwinism as actual evolution is problematic for several reasons. (i) It does not account for the generation of post-developmental variation, now known to act on circuitry throughout the whole of life, and on which selective mechanisms could act. (ii) We still miss an interpretation of heredity in terms of neurophysiology, so that the selected variants can be expanded and, from them, further variants be generated. In this way, the interaction between selection, variability and heredity can find the right spiking patterns to solve a problem [7]. (iii) Even granting selective stabilization of functional circuits (*sensu* Edelman) does not directly translate into preferential replication of said circuits.

The mechanisms for generating variability in neural spiking patterns are relatively simple to rationalize, and there are many works in the literature that take this aspect as a modelling objective [12]. But it is less obvious, of deeper implications and of far-reaching consequences to realize that a mechanism of ‘neuronal heredity’ between local complexes might exist. Thus, the copying of information of stabilized circuits into other circuits effectively results in selective dynamics. In our theory, the coaction of learning and selection is interpreted as the evolutionary dynamics of populations where the constituting individuals are neuronal circuits.

As explained above, heredity occurs when circuits that have reached satisfactory solutions transmit their contents to some other circuits that did not perform as well (figure 1). Although there is no replication of the population of neurons *per se* (as in a biological population), these repeated rounds of evaluation and replacement implement a mechanism of heredity [7,13] that is analogous to genetic inheritance. A neurobiologically realistic model for replication will be published elsewhere. For now, we emphasize that the physiological particulars do not affect the conclusions of this paper. We treat the component process of replication as a black box of which the content will be revealed later. Therefore, we perform an abstract analysis of evolutionary neurodynamics by linking basic theory in neuroscience and evolutionary biology under the assumption that neuronal heredity is solved. Note that the discussions on two mechanisms of accumulating knowledge (by evolution and by learning) have been largely isolated from each other. These two sides of the discussion are not exclusive. We of course recognize that spiking neurons, Hebbian learning and SSP exist, and are central components of cognition, but we argue that, on their own, they do not suffice for explaining how complex tasks are solved.

## 2. Models and methods

We note that on short time scales (milliseconds) spikes take place and the selective dynamics can act by rewarding different subnetworks of the neuronal circuit. Yet, variation in spiking can be produced due to changes in synaptic weights. On a larger time scale, SSP generates novel circuitry. For simplicity, we separate these two time scales. We first describe the joint action of learning and selection on several circuits by assuming that all circuits have the same topology of connections, but each one has a different spiking pattern. Later (in §2.4) we describe SSP.

### 2.1. Learning in parallel circuits

In the spiking models, learning occurs by updating the weights that determine the probability that a neuron fires. This update follows Hebb's rule, verbally stated as ‘neurons that fire together, wire together’. Hebb's rule has been modelled with fixed connection topology where the weights are allowed to change according to the covariance among neurons, as for example [14]2.1
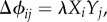
where *λ* is the learning rate, *X* = 1 if the neuron fires (on) and *X* = –1 if it does not (off), *ϕ_ij_* is the weight between neuron *i* and *j* and 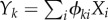 is the activity of neuron *k*, taken as a weighted sum of its input activity. (Note: in the neuroscience literature, weights are denoted by *w*; however, this notation is potentially confusing in the context of evolutionary analyses because a similar symbol is employed for fitness; table 1.)

**Table 1.** Analogy between the concepts in evolutionary genetics and neurodynamics.

evolutionary genetics	neurodynamics	symbol
loci/genes	neurons	*i*, *j*, *k*
no. loci	no. neurons in a circuit	*n*
alleles	neuron state (on/off)	*X*
allele frequency	firing probability	*ρ*
individual	neuronal circuit	*N*(=∞)
adaptive landscape	rewarding mechanism, score	*W*
mutation rate	switching probability	*A*, *M*
—	Hebbian weights	*ϕ*
—	learning rate	*λ*
—	synaptic cost	*k*

Note that when using this form of Hebb's rule, whenever a synapse exists between two inactive neurons *r* and *s*, the weights between these will strengthen, which is interpreted as an inhibitory process.

Hebb's rule is problematic because it allows weights to increase unboundedly. Thus, for computational convenience, we employ Oja's rule, which is a version of Hebb's rule with normalized weights,2.2
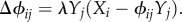


Oja's rule ensures that the weights are normalized, in this case with a Eucledian norm, so that 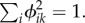


Whether any one neuron spikes or not is assumed to be a random event. The probability with which neurons change state (switch on or off) is given by an update rule *A* that depends on the state of the input neurons and their weights. Thus, the probability that a neuron *i* is on, 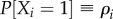, is given by the master equation2.3
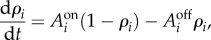
where 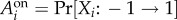 and 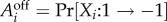 are the probabilities that inactive neurons spike and spiking neurons shut down, respectively. We assume that the update rule takes into consideration the state of both the focal neuron and the rest of the neurons in the circuit at the previous evaluation round. We also assume a time scale that is larger than the refractory period, so that spiking is only affected by the previous state of the network.

We assume that learning can be modulated more efficiently by allowing 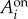 and 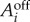 to have an effect on the network. Note that this description of learning is coarse-grained: it only tracks how often a neuron tends to be on as learning proceeds. This is a different view from that of machine learning, where neural networks are trained by a set of examples from which the weights are inferred. Then, from this inference, the model can be used to predict or classify data that were not included in the training set. Our goal in this paper is different: we consider parallel networks that try to solve a specific problem.

In solving a particular problem, we aim at minimizing the square deviation, 
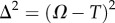
, between the target, *T*, and the output or solution, *Ω*. We assume that *T* is a given parameter and *Ω* is the output evaluation of the network. Since each network presents an alternative solution and has a different deviation Δ from the target, we minimize the mean value of the deviation, 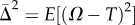. Under a proper scheme of neuronal network replication, this minimization amounts to Darwinian selection. First, each local network is weighted according to its fitness, given by *W* = exp[−*β*Δ^2^]. Second, circuits that have larger fitness are kept. Third, networks with lower fitness are overwritten with the content (spiking and/or weight states) of the circuits with large fitness. (There are several ways in which this copying can be implemented: this is the black box part as explained above.) Since in the present model we assume that there are infinitely many circuits, replacement need not be done explicitly: we simply consider the proportions of circuits (this is in order to have a direct link to classical population genetics models that assume infinite population size). Since we assume that copying is random across different neuronal loci, then the proportion of a circuit with a specific configuration is simply the product of the probability of the state of each neuronal locus (analogous to the Hardy–Weinberg assumption; [11, pp. 34–39]). Mathematically, we track the proportions, *ρ*, of active neurons and the distribution of weights across circuits. We consider (as a first approximation) an infinite number of circuits, each with a number *n* of neuronal loci. Thus, the proportion of active neurons at a locus corresponds to the probability of neuronal firing. Hence2.4
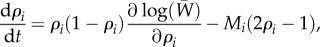
where the index *i* refers to the neuronal locus, *W_i_* is the fitness that a spiking neutron contributes to its circuit (which may be a function of the state of other neurons) and 
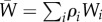
 is the mean fitness of that locus on the whole population of circuits. We refer to fitness as the amplification factor of the frequency of a given type (circuit). This fitness term, well known to evolutionary biologists, describes hill climbing in the direction of fitness increase [11]. We can approximate 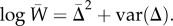 The second term represents the variability that is generated throughout learning. For simplicity, we assumed that the switching probability is symmetric (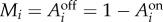), which is given by the activity rule2.5
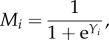
where 
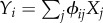
 is the activity or current of the focal neuron *i* (integration of the current of input neurons *j*), and *ϕ_ij_* are the weights determining the associations among neurons, which evolve according to Oja's rule. As more spiking neurons are connected, the activity of the focal neuron increases and its switching probability decreases asymptotically to zero. Whether a neuron stays on or off however depends non-trivially on the collective success of reaching the target *T*.

The electronic supplementary material, S1, shows that to first-order approximation we can track only the mean weight at every synapse and apply a general learning rule to all the average activities of the ensemble of circuits. That is, we approximate that each network has, on average, input activity 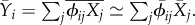 Since *X* = 1 with probability *ρ* and *X* = −1 with probability 1 − *ρ*, 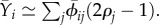 Although the variance of *X* is not zero, we neglect variance and correlation terms (they are small) in the average activity. However, even under these assumptions, each circuit can have a different spiking pattern from other circuits. Below we show that, even under these simplifying assumptions, learning has a dramatic effect by accelerating convergence to maximum fitness (or minimum 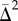). We will assume small initial values of the synaptic weights. Moreover, the variance of these becomes increasingly small as the neuronal complexes converge to a solution. Thus, we will make no further distinction between 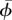 and *ϕ*.

#### 2.1.1. Numerical solutions to the neuronal dynamics

We numerically solve the system of *n* + *k* coupled ordinary differential equations to obtain the time evolution of a complex of multiple circuits (effectively infinite in number), where each has a fixed number *n* of neurons (equation (2.4)) and *k* synaptic weights (equation (2.2)); the latter depends on the connectivity of the learning network, which we assume to be undirected. Therefore, the weights are not symmetric. The initial conditions for the spiking and learning equations are random deviations from a uniform distribution. The system of equations is solved numerically for *t* = 10 000 time units, which ensures convergence for all parameters used. This time scale is considered to be of the order of approximately 10 ms. All simulations were implemented and solved in Mathematica v. 9.0 and/or v. 10.0.

### 2.2. Random networks

The learning network topologies were generated by drawing random graphs from three classes of distributions. First, the Erdős–Rényi model (ER), where nodes are connected randomly, assumes a fixed number of nodes *n* and certain probability *r* that each node is connected to any other node. Second, the Barabási–Albert (BA), famous for its scale-free properties, employs two parameters that control the network topology: the fixed number of nodes *n* and number of vertices *k* that are preferentially attached to each node. Third, the Watts–Strogratz (WS), or small-world networks, takes as parameters *n* nodes and a probability *r* of rewiring a vertex among two nodes in such a way as to avoid loops. We allow neither multiple edges nor more self-connections. These network models are built in Mathematica and employed as indicated in the software's Documentation Centre.

### 2.3. Information content of a synapse

How to measure the information content of a synapse is not obvious [15]. For our purposes, we employ mutual information, *H*, which describes the interdependency among two specific neurons in the context of a specific complex2.6
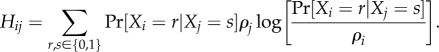


To calculate the conditional probabilities, we first evaluate the conditional activity *Y_i_*_|*j*_ of neuron *i* by fixing the value of neuron *j* to *ρ_j_* = 0 or 1. This gives a conditioned value of the switching probability, *M_i_*_|*j*_. The solution to equation (2.4), using the conditioned switching probability *M_i_*_|*j*_, gives the desired conditional probabilities. In this case, we keep the weights constant because we are only assessing the information capacity of the specific synapse and not the information capacity of the whole network. The exact expression of *H* is derived in the electronic supplementary material, S2, where we also show that for Gaussian selective landscapes *H* is approximately:2.7
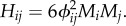


Mutual information quantifies how likely it is that, if one neuron spikes, the other one will also do so. If the neurons are not connected, *ϕ_ij_* = 0, implying *H_ij_* = 0. If the focal neuron *i* spikes randomly (large *M*), then the information content is low (in that case, *ϕ_ij_* is expected to be close to zero). Since 0 < |*ϕ_ij_*| < 1, the quadratic term dominates over *M*, making *H_ij_* proportional to 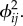 Hence, the information of a synapse increases as the weight increases. Note that for a given switching probability, the learning weights are higher in sparse networks than in fully connected ones. Thus, for any given synapse, the former can encode more information than the latter. This is partly because the weights are normalized: the relative weight of a synapse that connects a neuron with high degree is lower than the relative weight of a synapse connected to a neuron of low degree.

### 2.4. Structural synaptic plasticity

We implement SSP on a time scale much slower than that of associative learning. The system described above is in terms of statistical averages and can be regarded as conditioned on a given network of connections. We assume that synaptic connectivity changes occur in one arbitrary circuit (explained below). If the new topology improves fitness, it spreads across all circuits. For simplicity, this is implemented through a Metropolis–Hastings algorithm. That is, if fitness increases with the new topology, this spreads to all circuits. If fitness remains unchanged or decreases, then the layer might spread with probability exp(*W*_new_ − *W*_old_). Allowing for this fitness decrease facilitates the escape from states of impasse. Table 2 presents the algorithm we employ, and in the following paragraph we describe in more detail the implementations.

**Table 2.** Algorithm for SSP.

initialize:
*ρ* = random i.c.
*Φ* = random graph
If 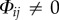
set *ϕ_ij_* = random
*W* = −∞
Do *M* times
Rewire 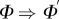
If new edge 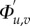 is added, set *ϕ_u,v_* = random
Numerically solve dynamics 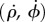 to equilibrium to get 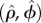 for all loci
Evaluate fitness 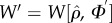
Metropolis–Hastings: with Prob 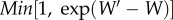 set
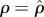
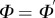
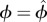
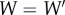
End

We assume that changes in synaptic structure follow two heuristic rules inspired from neuroscience. First, if two neurons are unconnected but they are highly likely to spike, then a new synapse among them can be introduced. There is evidence that synaptic rearrangements result from circuit rewiring upon (e.g. in neocortical pyramidal neurons) stimulation [16–18]. Algorithmically, we randomly choose pairs of neurons *i* and *j* with a probability 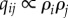 among the set of unconnected pairs of neurons, so that neurons that do not co-fire tend to be disconnected. Second, we allow existing synapses to be disconnected randomly with probability2.8
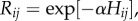
where *H* is the synaptic information (equation (2.7)). That is, if a synapse is informative, then it is unlikely to be disconnected, whereas if it contains no information, it is likely to be disconnected [3,19,20].

Third, we also allow random rewiring (irrespective of firing probabilities) with a small probability *u* = 0.01: we randomly and uniformly choose a connected pair *i*, *j* and eliminate the edge, and at the same time choose an unconnected pair *l*, *m* and establish an edge. In each time step, any (including all) of the above events are allowed to happen. Once the networks have been rewired, a new round of learning is performed. Initial conditions may or may not be modified (see Results). After a new equilibrium is reached, the new fitness is compared with the fitness before the rewiring. We additionally impose a multiplicative fitness cost per synapse of exp[–*kd*], where *k* is the penalty of each edge in the network and *d* is the number of edges of a given network. We run the simulations long enough to allow convergence to equilibrium.

## 3. Results

### 3.1. Selection and learning together speed up finding solutions

**Figure 2.**
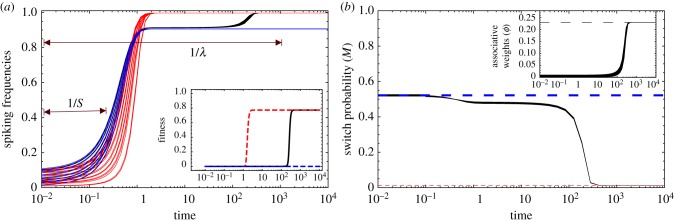
Example of selection-learning dynamics. (*a*) Selection-learning dynamics (black lines) compared with standard mutation selection with naive switching probabilities (*M* ∼ 1/2; blue) and with the run with the switching probabilities already learnt (red). Note that initially the blue and black lines overlap. Inset: evolution of fitness. (*b*) Evolution of the switching probabilities. Inset: evolution of Hebbian weights. *n* = 20, *S* = 5, *λ* = 0.001. Initial conditions for spiking probabilities and for initial weights are randomly sampled from a uniform distribution *U*[0, 0.01]. The learning network is fully connected. Note the log time scale.

To understand how learning and selection jointly act, we first assume a directional selection scenario, i.e. simple hill climbing where we target for all neurons to spike. In this case, fitness is given by 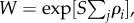 which has a constant gradient, 
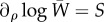
, for every neuronal locus. (This case can be seen as a limit where the target *T* is far from the current state, thus *S* = 2*βT*, so that selection acts mostly on the average distance to the target; later, we consider the variance term.) We assume that Hebbian learning is slower than selection; i.e. *λ* < *S*. This regime describes the coupling of learning with copying across circuits. Otherwise, learning would be independent in each circuit, associating random spikes and unable to learn the relevant features of the landscape, effectively acting against hill climbing. However, if learning is slower than selection, fitness increases the representation of the best solutions, and only once these are stabilized can learning create meaningful associations. Figure 2 presents a typical outcome where the process is characterized by three stages. We first observe an initial exponential increase in the proportion of active neurons. Compared with systems that do not learn, the dynamics are similar in the very early stages. This is because selection increases the representation of circuits that provide better solutions, but these are initially in very low proportions. These fitter solutions are simply products of lucky stochastic events. Initially, there is hardly any learning, indicated by light weights, and selective expansion simply amplifies those circuits that have higher activity. This amplification takes on the order of 1/*S* rounds of evaluation. As circuits become selected learning takes over, entering an incubation period where associations are built up because a good proportion of neurons fire correctly. Unlike the dynamics without learning that reach an equilibrium away from the optimum fitness (mutation–selection balance; blue line in figure 2*a*), with learning after the incubation period, associations are fully strengthened and the solution is finally reached, whereby switching probabilities reach a minimum (figure 2*b*). The width of the plateau has a duration of roughly 1/*λ* − 1/*S*. This regime is notable on a log scale. Although in absolute time, the selection process is so quick that it might pass unnoted, this selection stage is crucial to explore configurations that can be fixed through learning. We emphasize that this early stage corresponds to the selective stabilization in the Neural Darwinism theory.

Instead of favouring equally all neurons to spike, the landscape can be set to favour distinct neurons to fire preferentially over others. For instance, making 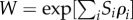 and allowing *S_i_* to take any arbitrary value introduces asymmetries to the landscape. Crucially, if the dynamics are re-run with the learnt weights, the equilibrium is reached orders of magnitude faster. We stress that this is true even if initial spiking probabilities are randomized (electronic supplementary material, S3).

For a given system, the associative weights increase (asymptotically) with the strength of selection (data not shown). We performed Spearman's ranked correlation test to measure the strength of the dependency. (Because of the nonlinearity, ‘standard’ Pearson's correlation is not a good measure for the dependency between *S* and *ϕ*.) In absolutely all cases, the *p*-values were numerically zero, indicating strong dependence among *S* and *ϕ* (data not shown). This strong statistical support indicates that the synapses encode the fitness gradient, directing variant spiking patterns accordingly: strong selection results in strong weights, which in turn decrease the switching probability. This leads to minimal variability of spiking, which maximizes speed of fitness increase. Conversely, weak selection leads to poor associations, resulting in large spiking variability, which allows exploration of the landscape.

We note that the learnt equilibrium point is independent of the learning rate *λ*. This turns out to be generally true, regardless of the fitness landscape. We also note that, under these ‘directional landscapes’, the initial conditions (of both weights and allele frequencies) do not affect the equilibrium state of the system. (However, later we will see that under more complex fitness landscapes this is not true.)

### 3.2. Formal analogy between evolutionary dynamics and neurodynamics

At this point, we formalize further the analogy with evolutionary biology, and more specifically with population genetics. First, we realize that the bimodal neuron model is analogous to a biallelic genetic system. We start by clarifying a small but crucial difference in the notation. While in the models considered in this paper neurons take signed states {–1, +1}, in population genetics alleles are typically denoted as {0, 1}. The +/− notation is convenient mathematically in order to describe Hebb's rules, thus, in our evolutionary analogy, we also require this property. Hence if *G* is the value of a gene or allele, then we define *X* = 2*G* – 1. In this way, we can readily apply the machinery from evolution to neuronal networks.

Second, we consider the spiking probability of a neuronal locus across all circuits (figure 1). This average, which is the probability Pr(*X_i_*) that a neuronal locus *i* fires in some of the circuits, is thus analogous to the average 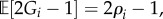 where *ρ_i_* are allele frequencies at locus *i*. Note that allele frequencies are interpreted as the probability of sampling a particular allele in the population. Thus, for the analogy to be consistent, population size needs to be analogous to the number of circuits involved in the learning. Although in both populations of individuals and of neuronal circuits, numbers are in fact finite, in this work we consider, as a first approximation, an infinite number. In this way, we do not need to worry about stochastic effects that complicate the analyses. However, we recognize that randomness due to finite population size (a.k.a. genetic drift) can play a crucial role in both evolution and learning. This is because randomness facilitates escaping local peaks and exploring the landscape in a less constrained manner. But before taking stochastic factors into consideration, we want to focus on the interaction between selection and learning in the infinite population model.

Third, upon reproduction, a population generates a new set of individuals, which sooner or later replaces the parental population. However, in neurodynamics, reproduction has to be interpreted in a particular way, because there is no generation of a new set of neuronal circuits. However, the selective copying into circuits with inferior performance effectively corresponds to a new population of circuits (figure 1*d*).

Given the analogies above, we can ask the converse question: what is the interpretation of the learning process in evolutionary dynamics?

Equation (2.3) describes the activity changes of neural networks across iterations, leading to an update rule of the spiking frequency of each neuron. In population genetics, this transition probability corresponds to a mutation rate. In molecular evolution, mutation rates are normally state-independent, dictated by, for example, copying errors of the polymerases that replicate DNA, repair mechanisms, or other molecular processes that do not depend on the genetic states of the individual or population. (Although there are genetic models that consider evolvable mutation rates; see Discussion.) The switching probability 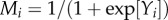 is dependent on the state of the system and follows directly from the update rule. Apart from this dependency, the equations (2.4) are analogous to a selection–mutation model. The resemblance is a natural outcome from the analogy laid out above.

But beyond the cosmetic similarity between the replicator–mutator equation and neural dynamics, the crucial difference is that the update rule is able to learn the local properties of the fitness landscape. By doing so, hill climbing towards a fitness peak is facilitated by generating variation directed towards the fitness increase.

### 3.3. Learning in rugged landscapes

We now consider the more complex adaptive landscape, given by 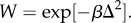 In evolution, these kinds of landscapes are known as ‘stabilizing selection’. The complexity of this landscape results from the nonlinear effects (epistasis in genetics and evolution). These are hard landscapes to explore because there are many local peaks or solutions, some equally optimal, some suboptimal, and simple hill-climbing algorithms often fail to converge to an absolute maximum of fitness.

**Figure 3.**
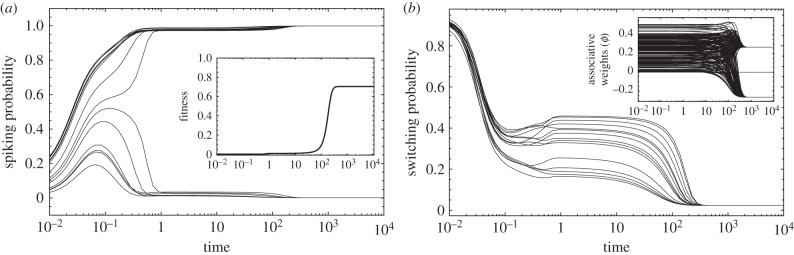
Neurodynamics in a stabilizing rugged landscape. Model parameters: *T* = 15; otherwise as in figure 2.

Figure 3 shows the neurodynamics. We find that exactly 15 neurons fire (with probability *ρ* = 0.995) and the remaining five remain off. In this case, the uninformative neurons are shut down. Which neurons spike and which do not is contingent on the initial conditions, but in this landscape the identity of each neuronal locus is meaningless. Different initial conditions can lead to different but equivalent solutions (data not shown).

### 3.4. Random and sparse topologies of the neuronal connections impair learning

So far we assumed that there are synapses among all pairs of neurons. Relaxing that assumption corresponds mathematically to fixing certain weights *ϕ_ij_* to zero, indicating that no synapse exists among neurons *i* and *j*. Under these circumstances, the equilibrium switching and spiking probabilities are more variable, with the spread determined by the connectivity of the underlying learning network. The electronic supplementary material, S4, presents some neurodynamic outcomes using different random topologies under directional and stabilizing landscapes. These topologies are drawn from different random graph models with various degrees (see Models and methods). We tried ER, BA and WS topologies. Each of these models has different statistical properties. Irrespective of these, there are two central conclusions. First, random networks lead to unfit solutions, where the systems cannot reach the target. This is true regardless of the target value, number of neurons and type of topology. The systems typically converge to a suboptimal solution where no further learning can happen and cannot escape local optima. We regard this as a situation where a network that was previously functional for another task is repurposed for a new task, and the initial topology is, regarding the new task, arbitrary. Thus, the initial circuit is not expected to be adapted to the new task. Consequently, what the system can learn is limited, and, in the vast majority of cases, suboptimal. We identify these solutions as states of impasse, i.e. there is no further progress possible because any small modification to the system leads to a lower fitness score.

The second central conclusion is that poorly connected neurons have very low input activity, leading to high switching probabilities. Highly connected nodes have small switching probabilities with spiking frequencies close to unity. Hence, only highly connected nodes (the less frequent) can learn efficiently. Since random topologies give suboptimal results, we consider that details regarding specific network distributions are secondary and discuss them only in the electronic supplementary material, S4.

Our choice of network distributions is arbitrary, motivated mostly by mathematical convenience; in principle, actual circuits might have different topologies [21,22]. Hence, the results above do not necessarily imply that brains are suboptimal unless fully connected. However, our results indicate that Hebbian learning does not suffice to solve complex problems in sparse networks (as are real neuronal complexes [16,22]) because it too often leads to impasses.

### 3.5. Structural synaptic plasticity

SSP is a mechanism that goes beyond the update of existing synaptic weights (i.e. Hebbian learning) by allowing new synapses to be established and old ones eliminated. This dynamical restructuring of neuronal circuits as the system learns has been shown to be important for the transfer of short-term to long-term memory [8]. However, we test the role of SSP in the more general scenario of problem and impasse solving.

Above we found that network topology impairs problem solving on complex learning landscapes. This is paradoxical because circuits in the brain are not fully connected, even when the type of connectivity is disputed and tissue-dependent. But our results do not rule out that there might be specific topologies that facilitate or optimize learning. We now show that, under SSP, the neuronal complexes form particular structures, which are unlikely to be recovered randomly, thus accounting for the negative results above.

The central hypothesis regarding SSP is that, by modifying the distribution of synapses, it provides new pathways to explore the space of solutions. This is why SSP can be an efficient mechanism to escape impasses.

### 3.6. Dependency on the number of neurons

**Figure 4.**
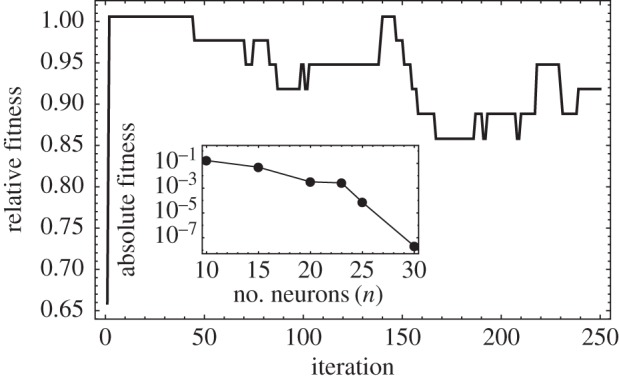
Dynamics of SSP. Example of a random realization with *n* = 30 neuronal loci (fitness is scaled to the maximum value). Inset: absolute fitness as a function of neuronal loci. Parameters: *T* = 7; otherwise as in figure 2. Synapses are assumed to have no cost.

SSP allows the guided exploration of the space of configurations, leading, on average, to an increase of fitness. Figure 4 shows that systems with few neurons evolve good solutions more easily than larger systems. This is clear: finding an optimal configuration with a few neurons requires fewer evaluations than larger networks simply because the search space of the former is much smaller than that of the latter. The number of possible configurations increases with *n*^2^, thus on the basis of trying one modification at a time, the convergence time increases nonlinearly with the number of neurons. However, although this holds true for our model, there is no reason to think that there cannot be parallel evaluations of different topologies in different complexes, dramatically alleviating this inefficiency. Note that one step in the iteration does not correspond to a physiological time unit because the Metropolis–Hastings algorithm only ensures convergence to the equilibrium distribution as dictated by detailed balance and considers no information regarding the diffusion leading to said equilibrium.

In each round of learning (i.e. after the system converged to equilibrium with a newly tested network), the current weights are being kept, and new connections are assigned to new random initial values. Alternatively, we can simply reset all weights to random values. The second strategy proves to be more efficient than the first, although it is not a necessary condition. It proved irrelevant whether spiking probabilities are reset or not (data not shown).

### 3.7. Structural synaptic plasticity leads to maximal connectedness

**Figure 5.**
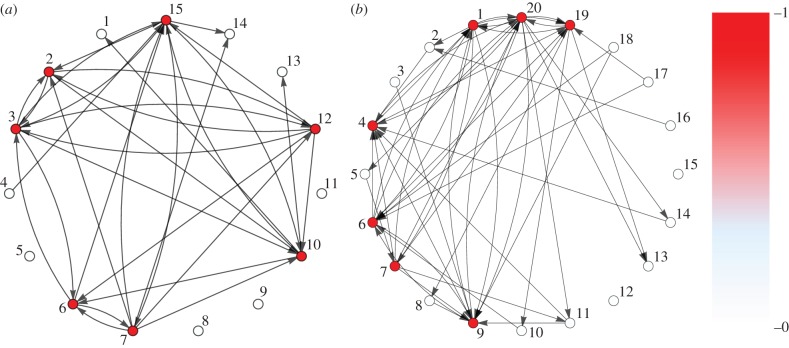
Evolved learning networks. Example of evolved networks with *n* = 15 (*a*) and *n* = 20 (*b*) neuronal loci. Darker nodes indicate higher frequency of active neurons. In both cases, the particular node labels are irrelevant, and the proportion of active neurons depends on the initial conditions and the history of the process. Note that, irrespective of the number of neuronal loci, the number of active components is correct. Parameters as in figure 4.

Because our test problem chooses for a target number *T* of spiking neurons, the optimal state has exactly *T* neurons on and the rest are off. Ideally, these *T* neurons are fully connected among them. We find that the complexes correctly converge to solve the problems, and the networks that evolve fully connect these active neurons (figure 5). In other words, the systems converge to networks that fully connect the required components to solve the problem.

The convergence to fully connected networks is due to two factors. The first is the need to switch on the right number of neurons, which requires strong synapses among them. The second is to switch off the unneeded components; this also requires connected components because negative weights between active and inactive decrease the probability of firing. If negative weights are not allowed, the system can only maximize fitness by ensuring the right neurons are on, and the networks converge to fully connect these components (figure 5).

### 3.8. Neuronal networks are robust to small costs of synaptic connections

**Figure 6.**
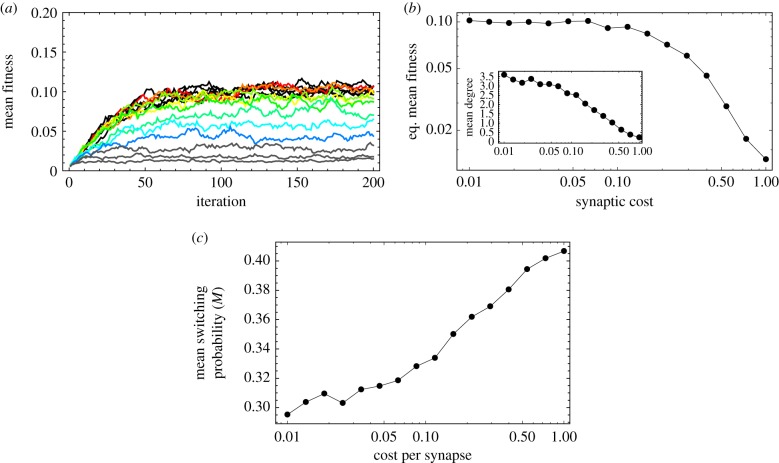
Neuronal systems under costly synapses. (*a*) Dynamics of the fitness (relative to the maximum) of neuronal systems under different cost per connection. Black curves on top: low costs (*k* < 0.05). Colour curves: intermediate costs increasing from *k* = 0.05 (red) to *k* = 0.5 (blue). Grey curves at the bottom: high costs (*k* > 0.5). Each curve is a replica of 77 independent simulations. (*b*) Equilibrium fitness as a function of the synaptic costs. Inset: average number of synapses of each neuron (network degree) against the cost per synapse. (*c*) Mean switching probabilities of the connected nodes against synaptic cost. Each point is an average at the stationary values (last 50 time points) and over the 77 simulations. Parameters: *n* = 10, *T* = 7, *S* = 10, *λ* = 0.01.

Now we penalize for the amount of connections that the networks have (figure 6). There are various reasons to assume this constraint. First, there are costs associated with synaptogenesis. Second, there are higher metabolic costs due to the transmission of action potentials, which increases at least proportionally (if not allometrically) with wiring. Third, there are major spatial constraints in the brain, limiting the amount of white matter that can be packed. In order to take into account these and other reasons for limiting the amount of neurons, we include a fitness cost to the system, exp[–*kd*], where *k* is the cost per synapse, and *d* is the number of synapses (number of edges in the learning network).

In figure 6, we observe that finding the solution to a problem is impaired as the cost of establishing synapses is increased. (In these examples, we target *T* = 7, but the particular choice of the target value is unimportant; in the electronic supplementary material, S5, we present results for other target values). Clearly, this is because the number of connections decreases with increasing cost, which in turn compromises spiking specificity.

It might be unsurprising that the number of synaptic connections decreases with their cost, and, naturally, the networks become less discriminative as they lose connections. However, they show a notable level of resilience (graceful degradation), because even when performance is impaired as synapses are eliminated, the required number of connected neurons is robust to the cost. In other words, as the cost increases, the networks still converge to structures that connect (even if sparsely) the necessary neurons (figure 6*b*). The networks lose performance as they lose synapses because neurons receive less input and therefore their switching probability becomes higher. Nevertheless, they tend to remain connected with as many components as possible.

In the stationary state, the distribution of networks is broad. Figure 6*b* shows that the average number of synapses decreases with the cost; this is also true for its variance (in the electronic supplementary material, S6, we present the degree distributions). As the cost increases, each neuron establishes fewer synapses with other ones. This is indicated by the notion of connectivity (figure 6*b*), i.e. the number of synapses that we need to remove to separate the network into two unconnected subsets. Typically in the evolved networks, low connectivity is due to a single poorly connected network, rather than to connected subcomplexes interconnected by a few synapses. As costs are very high (*k* ∼ 1), the networks are sparse and have several unconnected components.

We point out two important differences between random networks and the evolved distribution of networks. First, taking the random network as a null model (ER is the one that best matches the evolved distributions; electronic supplementary material, S6), we expect a binomial distribution *B*[*n* – 1,*p*]. The observed distributions are reminiscent of the binomial using the empirical *p*s. However, in all cases, we rejected the null hypothesis (*χ*^2^ tests, all *p*-values numerically zero); the expected variances are too low.

Second, despite the variability in the distribution of the evolved networks, these solutions are not in states of impasse. With fewer synapses, the input activities of the neurons are lower, translating into larger switching probabilities (figure 6*c*). This does not reduce specificity of firing: still the correct neurons are more likely to fire in an idiosyncratic manner. However, there is more ‘background’ noise due to fluctuations. We could say that, for larger costs, neurons are still accurate, albeit less precise.

Due to the redundancy of optimal solutions, occasional major restructuring of the networks is expected to occur during the whole lifetime for two reasons. First, once a peak has been found, disbanding the principal connections leads to major function impairment (fitness decrease). Second, establishing new synapses that are potentially as good as the existing central ones is unlikely due to the costs of synaptic connections. However, we do find occasional major network restructuring. Because of the strong coupling among several neurons and synapses, if a major connection is destroyed, subsequent changes attempting to compensate for the failure result in even worse fitness. At some point, there is a restitution of the system when a new fitness peak is approached. These are properties of self-organized criticality [23]. However, it might also be that the stochastic behaviour allows a few circuits to shift from suboptimal solutions to better ones, effectively jumping across fitness peaks. The subsequent replication of these successful solutions to other circuits can result in a full escape from impasse states. (Note the analogy with the *shifting balance* theory; [24,25].)

## 4. Discussion

### 4.1. Relationship to previous models

Using a Bayesian framework, Ullman *et al*. [12] proposed a model that explains aspects of cognitive learning in children. In their model, the brain implements a Bayesian update algorithm to form theories based on observed data. In their framework, theories map to a multi-dimensional landscape where well-formed theories lie at peaks. The dynamics include learning, but only as a local process. They argue that learning cannot account for the invention of new theories, but, rather, can only modify the degree to which we believe in any given theory. That is, learning acts to fine tune the theory around a peak. The proposal of new theories does not happen through learning, but through stochastic modification of the existing theories (in a data-independent manner, that is, there is random variation). If a new theory scores better than the previous ones, it is adopted with certain probability [26].

This model is similar to ours, particularly in the implementation. However, there is nothing mystical about this coincidence. What Ullman *et al*. and we describe belong to the Markov chain Monte Carlo class of models, which is a class of stochastic processes. One important common aspect is that learning alone does not produce any new configurations (networks in our case, theories in theirs), but only improves local adaptation given the current configuration. While their model describes processes occurring at a high level of cognition, we describe simpler processes at the neurophysiological scale. However, we reach similar conclusions regarding the need and limitations of learning in relation to other processes that can generate variant configurations that lead to a better performance. This coincidence and its consequences (see discussion in Ullman's paper) are preliminary evidence supporting our proposed physiological mechanisms.

Although Ullman *et al.* do not discuss the states of impasse, these are implicit in their models. That is, learning a local peak restricted to a given configuration results in weight values that always lead to lower scores if any modification is introduced. They resort to stochasticity as a means to jump across peaks. In our case, this stochasticity is introduced via SSP. There can be other sources of stochasticity, such as stimuli-dependent, chaos-induced [27] or finite-sampling effects, analogous to genetic drift. However, SSP not only is a component accounting for peak escape but is also known to be an important component of learning.

Note also the crucial difference in the search mechanisms in the two models. Kemp & Tenenbaum [26] use a greedy search algorithm on stochastically generated variation, whereas we adhere to the view of the Darwinian neurodynamics (the neuronal replicator hypothesis; [28,29]). The point is that, on vast combinatorial landscapes, an evolutionary search is known to produce impressive results. The greedy algorithm works for relatively small spaces but for larger spaces a more efficient search is needed [26]. It is remarkable that, although Ullman *et al*. [12] explicitly draw a rugged conceptual landscape, the possibility of an evolutionary search is not mentioned.

We call attention to a partly related model by Seung [10] invoking ‘hedonistic synapses’ that would release neurotransmitters stochastically, and an immediate reward would either strengthen or weaken them according to whether vesicle release of failure preceded reward, respectively. It was noted that such randomness in synaptic transmission would play the role of mutations in a Darwinian analogy. Seung also notes that stochasticity in action potentials could play a similar role and that that mechanism would be faster [30]. But since ultimately these mechanisms operate on a fixed topology, the limitations without SSP remain. Note that ‘copying’ in our mechanism is a fast component, intermediate between spikes and HP. This is a valid assumption if we assume that copying is aided by dedicated adaptations (cf. [31]).

### 4.2. The expansion–renormalization model

As mentioned in the Introduction, the ERM [8] assumes the generation of variant circuitry, which results in accelerated learning. Kilgard correctly points out that previous Darwinian frameworks do not take into consideration mechanisms of variation. He accounts for such variation in a verbal model. Unlike Kilgard's work, we assume specific neuronal rules, namely SSP, as the basic process that allows circuits to be modified. We have assumed two principal means for variation of the network structures: establishment of new synapses and disbanding of old synapses. The specific neurophysiological processes that facilitate rewiring among two arbitrary neurons are unknown. In this respect, we have introduced a novel idea. That is, we propose that the disbanding probability decreases with the amount of local information. Moreover, we have shown that synaptic information is proportional to the square of the synaptic weights. This is an interesting result because it relates the mechanistic aspects to the intuitive notion of neuronal function. Together, these two mechanisms can determine the dynamics of variation after layer replication.

On this line, Fauth *et al*. [19] propose and analyse a model similar to ours for the distribution of synapses between two neurons, by studying the interplay between Hebbian learning and SSP. They assume a constant rate of synaptogenesis for unconnected neurons, unlike the Hebbian-like mechanism we employ. Synaptic disbanding occurs with probability 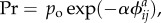 where *p*_o_, *a* and *α* are positive constants, which is of similar form to our *R* (equations (2.7) and (2.8)). Although in their case this form is not motivated by information content, they do point out that the topology of the network might constitute the basis of information storage and the role that this storage has in memory.

Kilgard's ERM hypothesizes that there must be a transient increase of circuitry variability (expansion) with a subsequent pruning of suboptimal synapses, reducing the variation (renormalization). We have not seen evidence for this. Rather, we find an increase in circuitry variability, with an eventual stabilization, but with persistent fluctuations and occasional ‘avalanches’, which afterwards recover and re-establish the network functionality. However, we think that the disparity between Kilgard's model and ours is superficial. The expansion might occur under specific fitness landscapes and might thus be problem-dependent. In our case, the fitness landscape has many equivalent maxima allowing for equally good solutions, with no force that generates excess variability. However, certain types of nonlinearities in the fitness landscape can certainly lead to that behaviour. Note that, in our implementation, we only allow for one circuit modification at a time (as Fauth *et al*. [19] do). The weights and topology of this circuit might be either copied to all circuits or discarded altogether. If many circuits can develop different circuits in the same evaluation round, we might find the expansion phase. (In fact in evolutionary models that allow for high mutation rates, there can be a transient increase in genetic variability, which is equivalent to the expansion phase of the ERM; e.g. [32]). Finally, it may well be true that new synapse formation is adaptive in that its rate is increased by the appearance of novel tasks, for which there is some evidence [1,21,33,34]. This provocation-based mechanism would easily lead to the expansion phase.

### 4.3. Size of the neuronal complexes

The complexity of the brain is reflected by the dimension of its constituent cells (billions of neurons) and by the intricate number of synapses (of the order of trillions). In some way, this accounts for the cognitive capacities of humans, although how is not fully clear. We have presented a hypothesis that serves as an organizing principle for this complexity. However, we have considered systems that employ as few as 10 neuronal loci on each layer. Although this small number is partly motivated by computational convenience, there are reasons to think that each circuit might not require an excessive number of neurons. First of all, it is well known that the brain is highly modular, with different neuronal complexes allocated to specific functions. We believe that some of these modules might be specialized for processing information in the way we propose, and, thus, are expected to be sub-structured into smaller functional complexes, each of which is constituted by a system of interconnected circuits acting in parallel and competing to solve tasks. Thus, the actual number of neurons dedicated to any given task depends on the number of circuits that are recruited for processing a given input, not just on the number of neuronal loci. Second, modular networks also facilitate rewiring because finding the right network configuration becomes increasingly harder for larger numbers of neurons. Hence, for an efficient implementation of SSP, brains might work in a modular way to facilitate rewiring of small complexes. Third, most complex tasks are likely to be split into subtasks, each of lower complexity employing relatively small circuits. In this divide and conquer strategy, smaller circuits can in turn be included in larger complexes to accomplish more elaborate tasks.

### 4.4. Information storage in neuronal circuits

Understanding the relationship between information capacity and synaptic changes is central in order to understand learning, memory and other aspects of cognition [3,16]. Under Hebbian learning, information storage relies solely on the modification of synaptic weights and is contingent on the existing connections. In the SSP scenario, the information capacity of neuronal complexes is adjusted through modification of the connections [20].

In our model, information content is stored not in the activity of neurons, but in the synaptic weights and switching probabilities. This has two important implications. First, this suggests that the loci of memory are circuits (not neurons), even though the mapping between memory loci and cognitive functioning might be mediated through the coordinated spiking of individual neurons (cf. [9]).

Previous findings also support the notion that information is stored in circuits, not neurons [20,35]. Furthermore, at a higher cognitive level, it has been proposed that consciousness can be gauged through information integration measures between neuronal complexes [15]. At a phenomenological level, it is well known that SSP at the level of both spine growth and modifications of the synaptic networks is directly induced by sensory experience [16,18] or by manipulating neuromodulators [17]. Despite these lines of evidence, which are compelling for our theory, we still require and lack direct experimental verification regarding the minimal complexity in the circuit distribution that results from solving particular tasks.

The second important implication is the procedural relationship between learning and variability. Even when these two are not the same and they constitute fundamentally two different processes, we have shown how learning fine-tunes the generation of variability. At the synaptic level, Hebbian learning modifies switching probabilities, which are the mechanism for generating variability in spiking. At the level of circuitry, SSP dictates longer term changes, where informative synapses persist and uninformative synapses are disbanded. Altogether, these two processes mediate the exploration of the complex combinatorial space by generating the required variability, guided by learning. These are the ‘fuel’ for the motor that results in effective changes, which are, ultimately, selection mechanisms.

### 4.5. Towards replicative neurodynamics

In this article, we report a crucial synergy between learning and fitness climbing, strengthening previous, related findings [13]. We have used simple models to show that the combination of evolutionary dynamics and learning in populations of neuronal networks is an extremely efficient one. Moreover, we also showed the relevance of SSP in this context: modifications of the topology of networks. However, there is another open possibility that can result from a combination of HP and SSP. We showed that certain networks are in general terms more efficient learners. Thus, the recruitment of an existing, efficient network which belongs to another task or population could in principle lead through SSP to the copying of its structure in the current network. In an analogous way to that in which DNA is copied, an existing network could replicate and such a structure would spread, allowing for problem solving. This has been previously proposed as the *neuronal replicator hypothesis* [13,28,29,36]. Since this idea is very recent, there has still been no experimental verification. But in this article, we advanced the mechanisms that justify the neural replicators. It remains open to study how the copying of the network topologies can occur.

Related to the issue of exponential strengthening versus exponential replication, the path evolution algorithm by Fernando *et al*. [37] is a remarkable suggestion. In that model, neurons along a path are assumed to code for some behaviour. While neuronal activity is fixed, paths grow collaterals and thus recruit new nodes. Neuronal activity can spread along different paths probabilistically and these paths can be evaluated and compared according to some performance (fitness) measure. Good paths become strengthened by reward, whereas bad ones are weakened. Various paths can have few or many common neurons. This algorithm explicitly incorporates SSP and selection and, despite the differences, is thus the closest precedent to our model. We improve on that model in two respects: we present a mathematical framework (in addition to simulations) that takes the first steps to unite theory of learning with that of natural selection, and we consider recurrent networks that posed a special problem for path evolution.

### 4.6. Mutations and recombination as creative sources

We should call attention to two possible usages of the term ‘mutation’ in the neuronal context. One we have seen before: stochasticity in firing or transmitter release. Another one is SSP itself: the term ‘synaptic mutation’ was coined by Adams [31] in this latter sense. Adams, by the way, foresaw the potential importance of the phenomenon for the performance of the nervous system. Note that Fernando *et al*. [37] in their path evolution model use SSP to implement ‘crossover’ between different paths.

Although visually reminiscent of genetic recombination, the synaptic mutations and the path crossovers are not formally equivalent to DNA crossover. In genetics, recombination does not create new allelic variability (on/off probabilities). Instead, it reshuffles the existing variants at any given locus. This certainly results in a ‘macromutation’ at the phenotypic level, but the genetic variability of the population remains intact. Thus, recombination does not increase allelic variation, but it does increase variation across circuits. The distinction is important in the context of our work: we assume the equivalent to free recombination. Namely, at any given neuronal locus, the copying can occur from any other circuit, irrespective of the state of the other neuronal loci. This provides the highest rate of reshuffling and is thus ‘creative’. The contrary limit is when only the complete content of a selected circuit can overwrite an out-selected circuit. This is an ‘asexual’ limit in that there is no recombination. The latter provides the fastest selective response, but is less creative in that it has no combinatorial power that exploits the extant variability.

### 4.7. Levels of selection

An exciting question is about the possible scope of multi-level selection (MLS; [38]) in this framework. First, one could go along with the idea of Adams [31] that synaptic strengthening is a kind of replication process. If so, selection on circuits already qualifies as MLS. Apart from this notion, we mention hierarchical reinforcement learning, which from a neuronal Darwinistic view must be MLS—an idea to pursue in the future. This is likely to be the case for high cognitive functions, such as language acquisition, where rules at higher and lower levels must arise and ‘coevolve’ to ensure communicative success.

### 4.8. Relationship to evolvability

Adam's synaptic mutations and Fernando's path crossover are analogous to modifications in the architecture of traits. This is a more powerful type of macromutation because it truly modifies the decoding of the information stored in neuronal states, in an equivalent way to development decoding the genetic information into a trait, which is one of the fundamental aspects of evolvability [39–41].

Evolvability is understood as the potential of a population to respond to selection and generate adaptive variation. How fast the response to selection is depends on the amount of genetic (or heritable) variation that can be produced. This can be given by standing variation, cryptic variation (due to epistasis, for example) or by mutational variance [42]. Although high mutation rates will provide source ‘material’ to respond to selection, these will also create load that keeps the population maladapted, overall limiting adaptation.

In our model, the optimal scenario is achieved if mutation rates can be increased as selection is started, and tuned down once the population approaches adaptation. Of course, genetic systems do not have a learning mechanism as the brain does. Nevertheless, there can be analogous processes [43].

We want to bring our analogy with evolution further and interpret the input current *Y* as a quantitative trait, with the weights *ϕ* taking the role of additive effects. This makes switching probabilities equivalent to evolvable mutation rates. These ideas are analogous to previous ones from quantitative genetics that consider mutational effects to be adaptive [44–46]. Although different from HP, modifier alleles for the mutational effects are selected indirectly, increasing mutation rates in the direction of the largest fitness increase [45]. While HP is analogous to mutations happening at a microevolutionary scale, SSP can implement more profound modifications that can be seen as equivalent to macroevolutionary changes and which take longer time scales in organismic evolution [47]. Even though SSP only uses local information to establish and eliminate synapses, the concomitant selective pruning of the networks also results in adaptive variation. Although acting at different evolutionary time scales in the field, brains implement both HP and SSP in a combined way to generate directed variation, and concomitant selection can even surpass the astonishing selective efficiency found only in animal breeding.

## 5. Conclusion

Understanding the role of plasticity in learning and cognition is one of the big goals of neuroscience. In this paper, we have addressed the role of plasticity from an evolutionary point of view. We complemented previous selectionist theories in neuroscience in a way that makes them formally analogous to evolution. This has led to formal analogies, enabling an evaluation of neuronal mechanisms in an evolutionary context. Our results indicate that selection can aid learning, strongly accelerating convergence to solutions. This implies that evolution might be more efficient within evolved brains than among organisms out in the wild. Also, selection acting on circuit variability can account for solving impasses (i.e. crossing fitness valleys). By considering known local mechanisms of neural plasticity, such as HP and SSP, we have studied how neuronal complexes can evaluate possible solutions in parallel, effectively competing to find optimal solutions. This in turn accounts for the distribution of circuits and is consistent with the principles of information storage and capacity of neuronal networks. Although we still rely on concepts that have not been experimentally proved to occur, such as mechanisms of neuronal copying, we have shown that the idea that the brain can implement evolutionary dynamics is feasible. Moreover, the consequences of such implementation are of great importance because they account for aspects which are still puzzling, despite the vast amount of knowledge from experimental neuroscience. The evolutionary view can bring a new perspective to understand some of these aspects.
